# Neoadjuvant chemotherapy using nanoparticle albumin-bound paclitaxel plus trastuzumab and pertuzumab followed by epirubicin and cyclophosphamide for operable HER2-positive primary breast cancer: a multicenter phase II clinical trial (PerSeUS-BC04)

**DOI:** 10.1007/s12282-022-01425-2

**Published:** 2023-01-07

**Authors:** Manabu Futamura, Kazuhiro Ishihara, Yasuko Nagao, Atsuko Ogiso, Yoshimi Niwa, Takumi Nakada, Yoshihiro Kawaguchi, Ai Ikawa, Iwao Kumazawa, Ryutaro Mori, Mai Kitazawa, Yoshiki Hosono, Masashi Kuno, Mana Kawajiri, Akira Nakakami, Makoto Takeuchi, Akemi Morikawa, Yoshihisa Tokumaru, Yasuo Katagiri, Yoshimasa Asano, Yoshinori Mushika, Toshio Shimokawa, Nobuhisa Matsuhasih

**Affiliations:** 1 Department of Breast Surgery, Gifu University Hospital, 1-1 Yanagido, Gifu, 501-1194 Japan; 2 Department of Surgery, Gihoku Kosei Hospital, Gifu, 501-2105 Japan; 3 Department of Surgery, Gifu Prefectural General Medical Center, Gifu, Japan; 4 Department of Breast Surgery, Gifu Municipal Hospital, Gifu, 500-8513 Japan; 5 Department of Breast Surgery, Asahi University Hospital, Gifu, 500-8523 Japan; 6 Department of Surgery, Takayama Red Cross Hospital, Takayama, 506-8550 Japan; 7 Department of Surgery, Gifu-Seino Medical Center, Ibi Hospital, Ibi, 501-0696 Japan; 8 Department of Breast Surgery, Central Japan International Medical Center, Minokamo, 505-8510 Japan; 9 Department of Pathology, Gifu University Hospital, Gifu, 501-1194 Japan; 10 Department of Surgery, Municipal Ena Hospital, Ena, 509-7201 Japan; 11 Department of Breast Surgery, Daiyukai General Hospital, Ichinomiya, 491-8551 Japan; 12 Clinical Study Support Center, Wakayama Medical University, Wakayama, 614-8509 Japan; 13 Department of Gastroenterological Surgery, Gifu University Hospital, Gifu, 501-1194 Japan

**Keywords:** Albumin-bound paclitaxel (Nab-PTX), Trastuzumab, Pertuzumab, HER2-positive breast cancer, Neoadjuvant chemotherapy

## Abstract

**Background:**

Nanoparticle albumin-bound paclitaxel (nab-PTX) is a promising antibody partner for anti-human epidermal growth factor receptor 2 (HER2). We performed neoadjuvant chemotherapy (NAC) for HER2-positive breast cancer (BC) using nab-PTX plus trastuzumab (T-mab) and pertuzumab (P-mab), followed by epirubicin and cyclophosphamide (EC).

**Methods:**

In this multicenter phase II clinical trial (January 2019–July 2020), patients with stage I (T1c)-IIIB HER2-positive primary BC were treated with four cycles of nab-PTX plus T-mab and P-mab, followed by four cycles of EC. The primary endpoint was the pathological complete response (pCR) rate. Secondary endpoints were clinical response rate (RR), adverse events (AE), and tumor-infiltrating lymphocytes (TILs) in biopsy samples.

**Results:**

In total, 43 patients were enrolled (mean age, 54 years). Twenty-two patients had HER2, and 21 patients had luminal/HER2-subtypes. The overall pCR rate was 53.5% (23/43, 95% CI: 42.6–64.1%, *p* = 0.184), whilst the pCR for HER2 was 68.2% (15/22, 95% CI: 45.1–86.1) and 38.1% for luminal/HER2 (8/21, 95% CI: 18.1–61.6%). The RR was 100% [clinical (c) CR:25, partial response (PR): 18]. AEs (≥ G3) included neutropenia (23.3%), leukopenia (7.0%), liver dysfunction (7.0%), and peripheral neuropathy (4.7%) when nab-PTX was administered. EC administration resulted in leukopenia (34.2%), neutropenia (31.6%), and febrile neutropenia (15.8%). The TILs in preoperative biopsy samples were significantly higher in pCR compared to non-pCR samples.

**Conclusion:**

Nab-PTX plus T-mab and P-mab induced a high pCR rate in HER2-positive BC, particularly in the HER2-subtype. Given that AEs are acceptable, this regimen is safe and acceptable as NAC for HER2-positive BC.

**Supplementary Information:**

The online version contains supplementary material available at 10.1007/s12282-022-01425-2.

## Introduction

Neoadjuvant chemotherapy (NAC) is widely used to cure breast cancer (BC), for both local control and for the eradication of micrometastases. In particular, it is essential for treating both human epidermal growth factor receptor 2 (HER2)-positive BC and triple-negative BC (TNBC) [1, 2]. Since the pathological complete response (pCR) rate has been suggested as a prognostic factor for non-luminal BC, we performed NAC to obtain a better pCR rate and develop a new treatment regimen [3]. The prognosis of HER2-positive BC has been significantly improved by the development of anti-HER2 therapy. In HER2-positive BC, several NAC trials have been performed. The NOAH study demonstrated that the addition of trastuzumab (T-mab) to combination chemotherapy significantly increased the pCR rate from 22 to 43% and prolonged event-free survival, which opened the window of NAC for HER2-positive BC [4].

The addition of trastuzumab (T-mab), lapatinib, and their combination to paclitaxel showed a 29.5, 24.7, and 51.3% pCR rates, respectively [5]. Furthermore, addition of T-mab, pertuzumab (P-mab), and their combination to docetaxel showed 31, 23, and 49% pCR rates, respectively [6]. Simultaneously, the CLEOPATRA study for metastatic HER2-positive BC reported the efficacy of dual HER2 blockade by T-mab and P-mab, suggesting that this strategy may lead to a cure for HER2-positive BC [7]. Several other clinical trials using a combination of T-mab and P-mab demonstrated a pCR rate of > 60% with anthracycline [8, 9]. These results indicate that the addition and/or combination of anti-HER2 therapy with taxanes can reveal synergistic effects and improve pCR rates. We thus understand the usefulness of dual blockade using anti-HER2 agents, even in early BC. In the Neosphere study, the pCR rate of the dual blockade by T-mab and P-mab was 18%. However, the combination of these blockades and docetaxel increased the pCR rate up to 49%, suggesting that the selection and addition of chemotherapeutic agents is important [6].

At present, there are three clinically available taxanes. Albumin-bound paclitaxel (nab-PTX; Abraxane^®^) is a solvent-free formulation of paclitaxel that reversibly binds to albumin. Nab-PTX was reported to be delivered to the tumor at a concentration 1.3 times higher than that of paclitaxel when administered at the same dose, resulting in a stronger antitumor effect in vitro [10]. Furthermore, nab-PTX was demonstrated to show higher clinical efficacy and cause hypersensitivity less commonly than paclitaxel or docetaxel [11, 12]. Based on these results, nab-PTX was administered as NAC in combination with an anti-HER2 agent. We previously reported a phase II study, named PerSeUS-BC01, for operable BC using nab-PTX. In that study, we found that the pCR rate for the HER2-subtype was 60% [13]. This result prompted us to further investigate the power of nab-PTX as NAC in Japan. Thus, we performed a meta-analysis of nab-PTX as NAC by collecting individual patient data from 16 phase II studies in Japan. In particular, the pCR rate for the HER2-subtype was 63.5% when combined with T-mab [14]. Therefore, to investigate the power and safety of nab-PTX with T-mab and P-mab followed by epirubicin and cyclophosphamide (EC) as NAC, we conducted a prospective phase II study called PerSeUS BC04 (Perpetual Study estimated-by the United Sections in Gifu for Breast Cancer 04). Our study aim is to demonstrate the efficacy of nab-PTX in combination with anti-HER2-therapy.

## Patients and methods

### Patients

This study was a multicenter, prospective, open-label, single-arm, phase II clinical trial that recruited patients through central registration. Women (age: 20–70 years) with histologically proven operable BC (T1c-T3N0-2M0, stages I–IIIB) were enrolled. Patients with a history of any previous therapy were excluded. All tumors were tested for estrogen receptor (ER), progesterone receptor (PgR), HER2, and Ki67 expression by immunohistochemistry (IHC) [15]. HER2 positivity was defined by an IHC score of 3 or 2 with gene amplification by in situ hybridization (ISH). HER2-subtype was defined by ER: J-score ≤ 1 or Allred ≤ 2 and luminal/HER2 by ER: J-score ≥ 2 or Allred ≥ 3 [16, 17]. Patients with bilateral BC, inflammatory cancer, active malignancy, active infection, or serious concomitant disease were excluded. Pregnant and lactating women were also excluded from the study. The Eastern Cooperative Oncology Group performance status of all patients was 0 or 1, and all patients showed adequate organ function [aspartate transaminase and alanine transaminase ≤ 3 times the upper limit of normal counts, bilirubin ≤ 2 mg/dL, creatinine ≤ 2 mg/dL, leukocyte ≥ 3000/mm^3^, neutrophil ≥ 1500/mm^3^, hemoglobin ≥ 9 g/dL, thrombocyte ≥ 10^5^/mm^3^, and normal left ventricular ejection fraction ≥ 50%)]. This study (UMIN 000,035,235) was approved by the local ethics committee or review board of each participating institution based on the Declaration of Helsinki. All the participants provided written informed consent.

### Treatment

**Fig. 1:**
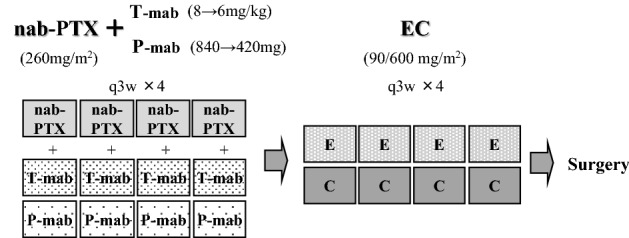
Schema for the study design. *Nab-PTX* nanoparticle albumin-bound paclitaxel, *T-mab* trastuzumab, *P-mab* pertuzumab. *E* epirubicin, *C* cyclophosphamide

The study design is illustrated in Fig. 1. Patients received four cycles every three weeks (q3w), nab-PTX (260 mg/m^2^) with T-mab 6 mg/kg (8 mg/kg as the loading dose) and P-mab 420 mg (840 mg as the loading dose), followed by four cycles of q3w EC (epirubicin: 90 mg/m^2^ and cyclophosphamide: 600 mg/m^2^); thereafter, surgery was performed. Fifteen minutes before nab-PTX with T-mab and P-mab to fifteen minutes after the end of nab-PTX infusion, frozen gloves and socks were used on both hands and feet [13, 18, 19]. Loxoprofen (3 tablets/day) and duloxetine (20 mg/day) were administered for 7 days from day 3. Each treatment was withheld for a maximum of three weeks only in cases of severe toxicity. The dose of each drug (EC and nab-PTX) could be reduced when febrile neutropenia (FN), grade 3–4 thrombocytopenia, or grade 3–4 nonhematologic toxicities occurred. The first permitted dose reductions were as follows: nab-PTX 220 mg/m^2^ and EC 70/450 mg/m^2^. The second dose reduction was nab-PTX 180 mg/m^2^ and EC 60/400 mg/m^2^ if severe adverse events (AEs) occurred after the first dose reduction. T-mab and P-mab were administered to all patients, except those who suffered from cardiac toxicity. However, in the case of FN, administration of granulocyte-colony stimulating factor (G-CSF) was allowed depending on the physician’s decision.

### Assessment of response and toxicity

The primary endpoint was pCR. The secondary endpoints were clinical response rate (RR), breast-conserving rate, assessment of tumor-infiltrating lymphocytes (TILs), Secreted Protein Acidic and Rich in Cysteine (SPARC) in biopsy samples, and safety. Clinical tumor response was assessed by the Response Evaluation Criteria in Solid Tumors (RECIST) version 1.1 using computed tomography or magnetic resonance imaging [20]. We defined pCR as the absence of histological evidence of residual invasive tumor cells in the breast and axillary lymph nodes (ypT0/is ypN0) [21]. Breast-conserving surgery (BCS) was performed when lumpectomy, quadrantectomy, or segmentectomy was appropriate. All the patients who received this regimen (more than one cycle of each regimen) were assessed for safety. Laboratory and nonlaboratory toxicities were evaluated using CTCAE version 5.0 [22].

### Pathological evaluation

We evaluated TILs in preoperative tumor biopsy samples using a standard methodology for the visual assessment of hematoxylin and eosin (HE) sections. In brief, paraffin-embedded biopsy samples (4–5 μm) stained with HE were observed at a magnification of × 200 by experienced pathologists. The average TILs were counted from five different views surrounding each tumor according to the instructions of an international TILs working group [23]. As SPARC is suggested to be associated with tumor progression and a predictive marker of nab-PTX, we also performed IHC for SPARC expression using biopsy samples, as described previously [13, 24]. Programmed death-ligand 1(PD-L1) was also evaluated using the SP-142 antibody, as previously described [25]. IHC analysis was performed by an experienced pathologist. The neutrophil-to-lymphocyte ratio (NLR) and platelet-to-lymphocyte ratio (PLR) were calculated using clinical data at the beginning of chemotherapy [26, 27].

### Statistical analysis

#### Sample size

In a previous study conducted in neoadjuvant settings, the pCR rate of HER2-positive BC for nab-PTX plus T-mab followed by EC was 45.5%; further, it was 29.4% for luminal/HER2 and 60% for HER2-subtypes [13]. The German group reported a 60% pCR rate for HER2-positive BC as a NAC by nab-PTX plus T-mab and P-mab followed by EC [28]. The required sample size was estimated based on a threshold pCR rate of 45.5%, an expected pCR rate of 60 and 90% power, and an alpha error of 0.10 (one-sided) using the binomial test. Given an estimated 5% of ineligible patients, the target sample size was estimated to be at least 39 patients. Furthermore, to evaluate exploratory variables (tumor size, nodal metastasis, subtype, nuclear grade SPARC/ PD-L1 expression, NLR/PLR, and TIL) for pCR, univariate analyses were performed using either the Fisher’s exact test or two-sample *t*-test. Statistical significance was set at *p* < 0.05.

## Results

### Patient characteristics

In this study, 43 eligible patients were enrolled from January 2019 to July 2020 as summarized in Table 1. The median age of the patients was 54 years (range, 28–69 years). Forty-one patients were diagnosed with invasive ductal carcinoma and two with mucinous carcinoma by core needle or vacuum-assisted biopsy. Tumor sizes were as follows: T1, 5 (11.6%); T2, 36 (83.7%); and T3, 2 (4.7%). Axillary lymph node metastasis was identified in 21 (48.8%) patients. Eleven patients (25.6%) had stage I disease, 15 (34.9%) had stage IIA disease, 13 (30.2%) had stage IIB disease, and 4 (9.3%) had stage IIIA disease. ER positivity was observed in 21 (48.4%) patients. HER2 positivity by IHC was classified as follows: score 3, 37 (86.0%) and score 2 with ISH-positive, 6 (14.0%).

**Table 1:** Patient chatacteristics

	Number of patients	%
Age, Years		
Median	54	
Range	28–69	
≥ 50	29	67.4
< 50	14	32.6
Performans status = 0, 1	43	100
Clinical tumor stage		
T1	5	11.6
T2	36	83.7
T3	2	4.7
Clinical nodal stage		
N0	22	51.2
N1	20	46.5
N2	1	2.3
Clinical stage		
I	11	25.6
IIA	15	34.9
IIB	13	30.2
IIIA	4	9.3
ER status		
Positive	21	48.8
Negative	22	51.2
HER2 status		
3 +	37	86.0
2 + , ISH +	6	14.0
Histological type		
Special type (mucinous)	2	4.7
Invasive ductal carcinoma	41	95.3
Tuble forming	6	
Solid	14	
Scirrhous	15	
Unknown or Mixed type	6	

*ER* Estrogen receptor, *PgR* progesterone receptor. *HER2* Human Epidermal Growth Factor 2. *SPARC* secreted protein, acidic and rich in cysteine

### Compliance and study completion

All patients who received at least one cycle of nab-PTX were included in the safety and response analyses. Thirty-eight (88.4%) patients completed all four cycles of nab-PTX with T-mab and P-mab. Four patients discontinued nab-PTX because of liver dysfunction, and one because of allergy. Dose reduction was required in seven (16.3%) patients, and dose delay was required in 12 patients (27.9%). Consequently, 38 patients received EC, 30 of whom completed the regimen (78.9%). Two patients discontinued EC owing to allergies and fatigue. For this, dose reduction was required in 12 patients (31.6%) and dose delay was required in 11 patients (28.9%). Finally, 33 of the 43 patients (76.7%) completed the entire regimen. All the patients underwent curative surgery after chemotherapy. None of the patients discontinued the protocol owing to protocol deviation.

### Clinical and pathological assessments

**Fig. 2:**
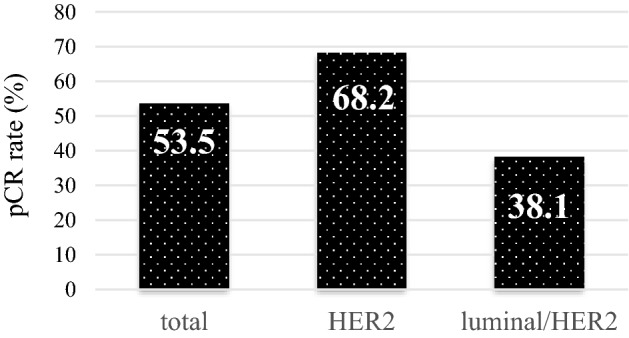
Pathological complete response (pCR) rate for all or each subtype of HER2-positive breast cancer. *pCR* pathological complete response, *HER2* human epidermal growth factor receptor 2

A pCR was observed in 23 of 43 patients (53.3%; 95% confidence interval [CI): 42.6–64.1), *p* = 0.184). The pCR rates for patients with the HER2 and luminal/HER2-subtypes were 68.2% (15/22, 95% CI: 45.1–86.1) and 38.1% (8/21, 95% CI: 18.1–61.6%), respectively (Fig. 2).

In addition, the clinical RR following completion of nab-PTX plus T-mab and P-mab was 92.8% (39/42).

**Fig. 3:**
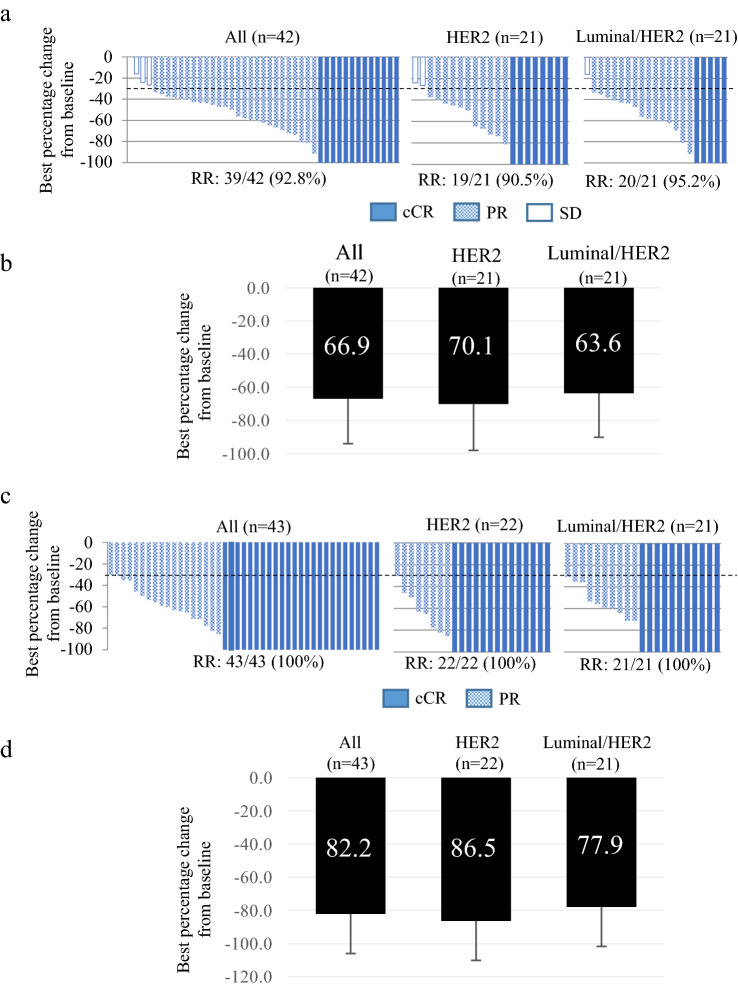
Response to nab-PTX therapy. **a** Waterfall plot to show the efficacy of nab-PTX with T-mab and P-mab. cCR, PR, and SD were indicated. **b** Best percentage change from baseline in all or each subtype after nab-PTX with T-mab and P-mab. The average rate with an error bar for the standard division is shown. **c** Waterfall plot to show the efficacy after EC. **d** Best percentage change from baseline in all or each subtype after completion of EC. cCR and PR were indicated. *Nab-PTX* nanoparticle albumin-bound paclitaxel, *T-mab* trastuzumab, *P-mab* pertuzumab, *cCR* clinical complete response, *PR* progesterone receptor, *RR* clinical response rate, *SD* standard deviation, *EC* epirubicin and cyclophosphamide

With the exception of one case because of unmeasurable lesions (*n* = 42), 90.5% (19/21) of patients had HER2 and 95.2% (20/21) of patients had luminal/HER2-subtypes (Fig. 3a). The best percentage changes from base line were 66.9% for all, 70.1% for HER2, and 63.6% for luminal/HER2 (Fig. 3b). The clinical RR after completion of EC increased to 100% (43/43) in all cases, including 100% for both the HER2 (22/22) and luminal/HER2-subtypes (21/21) (Fig. 3c). The best percentage improvements from base line for all, HER2, and luminal/HER2 were 82.2, 86.5, and 77.9%, respectively (Fig. 3d). No patient showed PD during the protocol treatment. All patients underwent the planned surgery, in which BCS was performed in 13 of the 43 (30.2%) patients.

### Safety profile

The incidence of AEs (all grades and grades 3/4) is presented in Table 2. During nab-PTX with anti-HER2 therapy, grade 3 hematological toxicities included neutropenia (23.3%), leukopenia (7.0%), FN (4.7%), anemia (2.3%), and liver dysfunction (7.0%); while grade 3 non-hematological toxicities included peripheral sensory neuropathy (4.7%), arthralgia (2.3%), myalgia (2.3%), and diarrhea (4.7%). Infusion reactions (grade 1/2) were observed in 11.6% of patients. Grade 3 hematological toxicities during EC therapy included leukopenia (34.2%), neutropenia (31.6%), FN (15.8%), anemia (2.6%), and liver dysfunction (2.6%), whereas grade 3 non-hematological toxicities included nausea (2.6%), stomatitis (2.6%), and sensory neuropathy (2.6%). Most AEs were controlled. G-CSF was administered to one of the three patients with FN. The incidence of non-hematological AEs, such as arthralgia, myalgia, and peripheral neuropathy, was lower during EC therapy than during nab-PTX therapy, suggesting that the AEs caused by nab-PTX resolved in a short period of time, as reported previously [12].

**Table 2:** Most common adverse events

Adverse events	nab-PTX (*n* = 43)		EC (*n* = 38)	
	All grades	Grade 3/4	All grades	Grade 3/4
Hematologic				
Leukopenia	16 (37.2)	3 (7.0)	20 (52.6)	13 (34.2)
Neutropenia	16 (37.2)	10 (23.3)	17 (44.7)	12 (31.6)
Febrile neutropenia	2 (4.7)	2 (4.7)	6 (15.8)	6 (15.8)
Anemia	11 (25.6)	1 (2.3)	14 (36.8)	1 (2.6)
Liver dysfunction	18 (41.9)	3 (7.0)	11 (28.9)	1 (2.6)
Nonhematologic				
Sensory neuropathy	33 (76.7)	2 (4.7)	21 (55.3)	1 (2.6)
Arthralgia	22 (51.2)	1 (2.3)	6 (15.8)	0
Myalgia	23 (53.5)	1 (2.3)	6 (15.8)	0
Nausea	17 (39.5)	0	28 (73.7)	1 (2.6)
Diarrhea	2 (4.7)	2 (4.7)	8 (21.1)	0
Constipation	11 (25.6)	0	14 (36.8)	0
Stomatitis	23 (53.5)	0	17 (44.7)	1 (2.6)
Dysgeusia	12 (27.9)	0	13 (34.2)	0
Infusion reaction	5 (11.6)	0	1 (2.6)	0

### TIL evaluation response to nab-PTX and SPARC expression

**Fig. 4:**
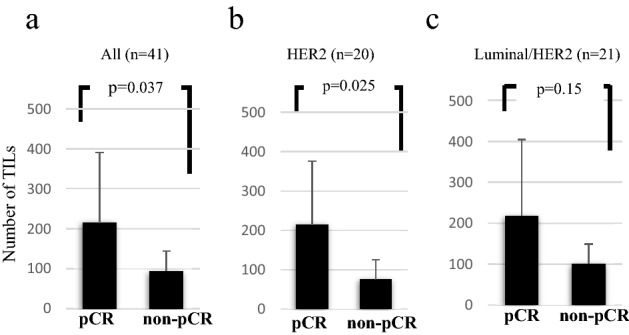
Number of TILs in biopsy samples. TILs counted using each biopsy sample before NAC were compared between the pCR and non-pCR groups. The average number of TILs with an error bar for the standard division is shown for all (**a**, *n* = 41), HER2 (**b**, *n* = 20), and luminal/HER2-subtypes (**c**, *n* = 21). *TILs* tumor-infiltrating lymphocytes, *NAC* neoadjuvant chemotherapy, *pCR* pathological complete response, *HER2* human epidermal growth factor receptor 2

We evaluated TILs in 41 patients whose biopsy HE slides were available. In these 41 cases, the number of TILs was significantly higher in pCR cases (*n* = 21, 214.8 ± 175.6) than in non-pCR cases (*n* = 20, 121.3 ± 84.3) (*p* = 0.037) (Fig. 4a). Particularly, the TILs in the HER2-subtype were much higher in pCR cases (*n* = 14, 213.3 ± 177) than in non-pCR cases (*n* = 4, 75 ± 58.6); however, no difference was observed in luminal/HER2 between pCR and non-pCR cases (Fig. 4b, c).

Next, we performed IHC for SPARC and PD-L1 in 34 patients for whom samples were available. As previously reported, SPARC expression was observed not only in tumor cells (*n* = 14, 14/34 [41.2%]), but also in stromal cells (*n* = 34, 34/34 [100%]) (Suppl. Table 1). SPARC expression in tumor cells was not associated with pCR. SPARC-positive tumors showed 62.8% shrinkage after nab-PTX plus T-mab and P-mab treatment, while SPARC-negative tumors showed 67.4% shrinkage (not significant). PD-L1 expression was positive in 13/34 (38.2%) patients (Supl. Table 2); however, it was not associated with pCR (Suppl. Figure 1a, b, c).

### Univariate analysis

To clarify pCR-related factors, we performed univariate analysis because the sample size was too small to perform a multivariable analysis. As shown in Table 3, TILs were independently significant (*p* = 0.037). Subtype, lymph node metastasis, tumor size, and NG, Ki67, NLR, PLR, and SPARC/PD-L1 expression were not significant factors.

**Table 3:** Univariate analyses

No		pCR		*p* value
		Yes	No	
		23	20	
Subtype	Luminal/HER2	13 (68.4)	10 (58.8)	0.069
	HER2	6 (31.6)	7 (41.2)	
Lynphnode metastasis	( +)	11 (47.8)	9 (45.0)	1.000
	( −)	12 (52.2)	11 (55.0)	
Tumor (mm)		3.69 (4.43)	2.91 (1.27)	0.453
Ki67		35.91 (13.78)	39.47 (14.77)	0.429
Nuclear grade	3	7 (38.9)	7 (43.8)	0.730
	1, 2	11 (61.1)	9 (56.2)	
NLR		2.64 (1.15)	2.15 (0.76)	0.117
PLR		177.39 (67.92)	154.31 (51.12)	0.221
SAPRC expression	( +)	7 (38.9)	7 (43.8)	1.000
	( −)	11 (61.1)	9 (56.2)	
PD-L1 expression	( +)	9 (50.0)	12 (75.0)	0.172
	( −)	9 (50.0)	4 (25.0)	
TIL		214.8 (175.6)	121.3 (84.3)	0.037

## Discussion

In this study of HER2-positive BCs, our protocol achieved a pCR rate of 53.5% (95% CI: 42.6–64.1%). The low threshold limit was below 45.5% and the *p* value was 0.184, which were not statistically significant. Although there was a clinically significant difference, the number of cases in our study was 43, with a power of only 37.0% at a significance level of *α* = 0.05. Thus, we needed 98 cases to obtain a significant difference. However, it is important to note that we obtained a pCR rate of 68.3% (95% CI; 45.1–86.1%) for the HER2-subtype and 38.1% (95% CI; 18.1–61.6%) for the luminal/HER2-subtype. Considering that our previous pCR rate data using nab-PTX with T-mab followed by EC demonstrated a pCR rate of 36.4% (8/22) for all HER2-positive BCs, 60% for the HER2-subtype, and 29.4% for the luminal/HER2-subtype [13], the addition of P-mab promoted a pCR rate up to approximately 10%. The pCR rate in the Neosphere study was 63.2% for the HER2-subtype by administration of docetaxel with T-mab and P-mab [7]. Furthermore, in the TRYPHAENA and BERENICE studies, it increased up to 65–81%, which was obtained not only due to taxane administration with T-mab and P-mab, but also due to the administration of additional anthracycline [8, 9]. In contrast, the pCR rate of KRISTINE was 73.2% for the HER2-subtype due to the administration of TCH (docetaxel, carboplatin, and T-mab) with P-mab [29]. Fasching et al. reported that the pCR rate could be improved by 20% with the addition of P-mab, even in routine clinical use [30]. These data suggest that dual blockade by both T-mab and P-mab is essential, and the selection of a combinational chemotherapeutic agent is also important for NAC against HER2-positive BCs. As shown in Fig. 3a and 3c, clinical CR and PR were 31% (13/42) and 92.9% (39/42) after nab-PTX; however, they reached 58% (25/43) and 100% (43/43) after EC, suggesting that EC after nab-PTX also plays an additional role in antitumor efficacy.

We have been interested in nab-PTX from the three available taxanes because it has several advantages compared with other potential drugs. Paclitaxel can improve immunological status by decreasing regulatory T cells (Tregs) and increasing Th1 cytokines, such as IFNγ-and/or IL-2, in vitro and in vivo [31, 32]. Nab-PTX can accelerate antitumor effects by activating M1 macrophages and MHCII/CD80/CD86, an important factor for activating helper T and naïve T cells [33]. Moreover, anti-HER2 antibodies, such as T-mab and P-mab, exhibit antibody-dependent cell-mediated cytotoxicity [34, 35]. Although the addition of immunotherapy revealed an apparent synergistic effect in a neoadjuvant setting for TNBC, this strategy was not effective for HER2-positive BCs, suggesting that at present, the selection of a chemotherapeutic agent is key for the success of NAC in HER2-positive BCs [36].

TILs were also a predictive factor for the nab-PTX regimen (Fig. 4). Although our data were obtained from patients with a triweekly administration of nab-PTX (because only triweekly use was approved in Japan), the GeparSepto study of weekly administration of nab-PTX with T-mab and P-mab to the HER2-subtype reached up to 76% of pCR rate [28]. These data suggest that nab-PTX combined with T-mab and P-mab can improve the intratumoral immunological environment and enhance the efficacy of HER2-positive BCs.

Nab-PTX also has a clinical advantage; namely, it shows a very low rate of hypersensitivity because AEs tend to bother patients, which often leads to the termination of the essential treatment. According to previous studies comparing conventional paclitaxel and docetaxel, nab-PXT demonstrated < 1% hypersensitivity of any grade without premedication with steroids [11, 12]. In the current study, allergy to nab-PTX (a suspicious case) occurred in only one case (2.3%), indicating that scheduled administration was performed. All other AEs were controlled. As shown in Table 2, PTX-specific AEs, such as sensory neuropathy, arthralgia, myalgia, and liver dysfunction occurred at a high frequency, whereas nab-PTX was stably administered in combination with anti-HER2 therapy by controlling the appropriate supportive care, leading to preferred clinical outcome [13, 18, 19]. However, according to data from a Japanese meta-analysis, the 5-year disease-free survival/overall survival after nab-PTX with T-mab plus anthracycline was 86.9/96.6% for HER2 and 90/97.1% for the luminal/HER2-subtype [14]. Furthermore, the GeparSepto study demonstrated a statistically significant elongation of disease-free survival in the nab-PTX group [37].

Our multi-institutional prospective phase II trial had some limitations. This was a single-arm study. The small sample size of each subtype may have influenced the results of the primary endpoint. Despite statistical analysis undertaken in a preplanned manner, the multivariable analysis was not performed because our sample size was not large enough.

In conclusion, we developed a new chemotherapeutic regimen for HER2-positive BCs. Nab-PTX in combination with T-mab and P-mab is a safe and effective treatment regimen, particularly for HER2-subtype BCs. The microenvironment of tumors, such as TIL, may be a predictive factor for anti-HER2 NAC therapy.

## Supplementary Information

Below is the link to the electronic supplementary material.

Supplementary Fig. 1 Evaluation of SPARC and PD-L1 in biopsy samples IHC for SPARC and PD-L1 was performed on 34 samples. a. SPARC expression in the tumor tissue was evaluated. There was no statistically significant difference between the pCR (n = 18) and non-pCR groups (n = 16). b. Best percentage change from baseline, depending on SPARC expression in the tumor. The average rate with an error bar for standard division is shown. c. PD-L1 expression in tumor and/or stromal tissues was evaluated. There was no statistically significant difference between the pCR (n = 18) and non-pCR groups (n = 16). IHC, immunohistochemistry; SPARC, Secreted Protein Acidic and Rich in Cysteine; PD-L1, programmed death-ligand 1; pCR, pathological complete response (XLSX 10 KB)

Supplementary file2 (PPTX 472 KB)
