# Supplementary material for: Neoadjuvant chemotherapy using nanoparticle albumin-bound paclitaxel plus trastuzumab and pertuzumab followed by epirubicin and cyclophosphamide for operable HER2-positive primary breast cancer: a multicenter phase II clinical trial (PerSeUS-BC04)
Source: Breast Cancer. 2023 Jan 7;30(2):293–301. doi: 10.1007/s12282-022-01425-2 (PMC9950177; doi:10.1007/s12282-022-01425-2)
Supplement: Supplementary file 2 — Supplementary file2 (PPTX 472 KB) [file 12282_2022_1425_MOESM2_ESM.pptx]

## Slide 1
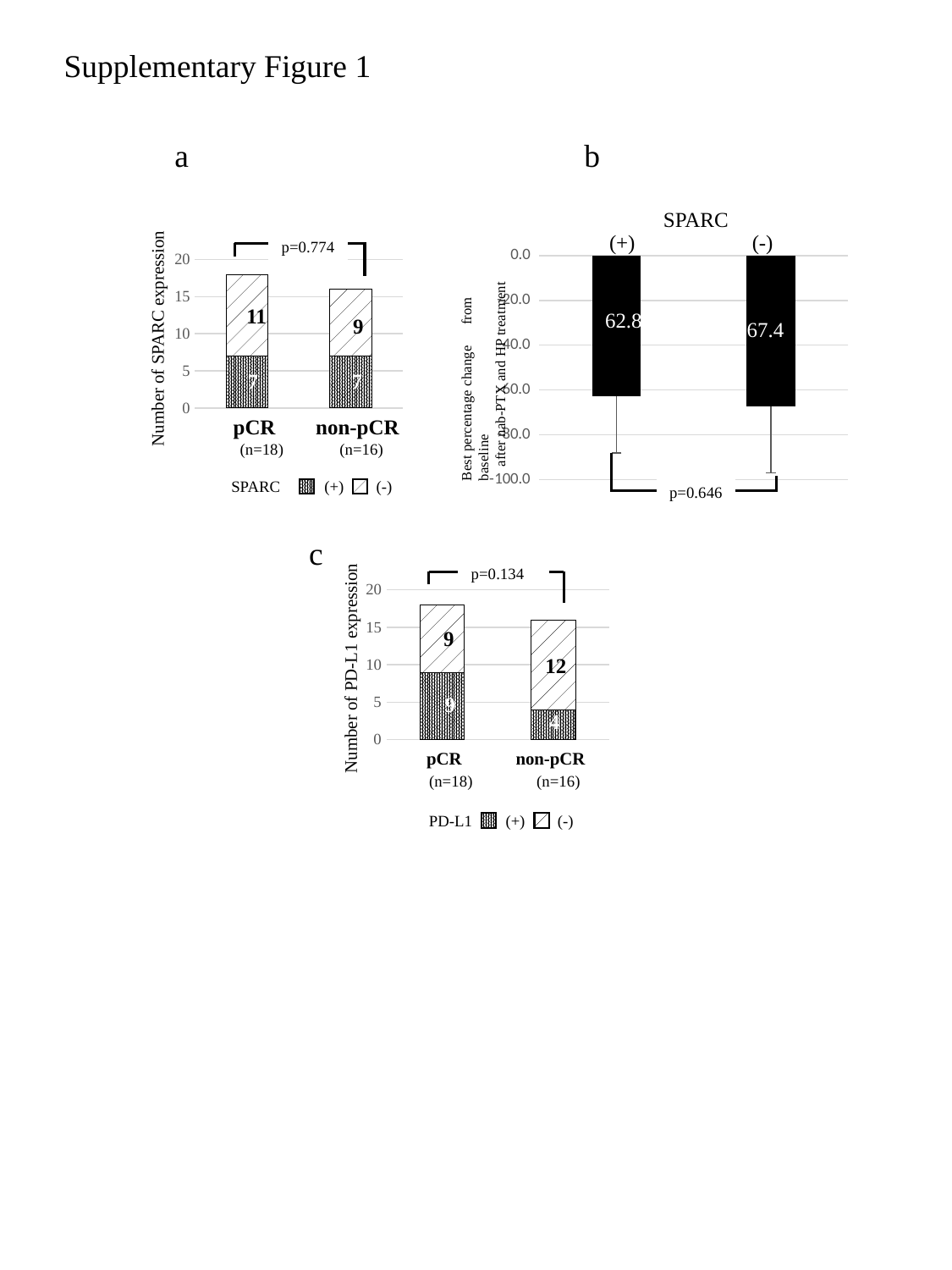

Supplementary Figure 1
a
b
SPARC
(+)
(-)
### Chart
| Category | |
|---|---|62.8
67.4
Best percentage change　from baseline
 after nab-PTX and HP treatment
p=0.646
p=0.774
### Chart
| Category | SP(+) | SP(-) |
|---|---|---|
| pCR | 7.0 | 11.0 |
| non-pCR | 7.0 | 9.0 |11
9
Number of SPARC expression
7
7
pCR
non-pCR
(n=18)
(n=16)
SPARC
(+)
(-)
c
p=0.134
### Chart
| Category | (+) | (-) |
|---|---|---|
| pCR | 9.0 | 9.0 |
| non-pCR | 4.0 | 12.0 |9
12
9
4
non-pCR
pCR
(n=18)
(n=16)
PD-L1
(+)
(-)
Number of PD-L1 expression
